# Social-Emotional Learning (SEL) and Its Impact on Teacher Stress, Self-Efficacy, and Attitudes Towards Inclusion: Longitudinal Insights from the StaFF-BL Project

**DOI:** 10.3390/bs15111511

**Published:** 2025-11-07

**Authors:** Dennis Christian Hövel, Patrizia Röösli, Ankica Jurkic, Melanie Nideröst, Pierre-Carl Link, Fabio Sticca

**Affiliations:** Institute for Educational Support for Behaviour, Social-Emotional, and Psychomotor Development (IVE), University of Teacher Education in Special Needs (HfH), 8057 Zurich, Switzerland; patrizia.roeoesli@hfh.ch (P.R.); ankica.jurkic@hfh.ch (A.J.); melanie.nideroest@hfh.ch (M.N.); pierre-carl.link@hfh.ch (P.-C.L.); fabio.sticca@hfh.ch (F.S.)

**Keywords:** social-emotional learning, teacher stress, self-efficacy, inclusion, behavioural screening, longitudinal study

## Abstract

Social-emotional and behavioural difficulties are among the most common developmental problems in childhood and adolescence and present substantial challenges for teachers and schools. Universal social and emotional learning (SEL) programmes combined with standardised diagnostic procedures have been proposed as a promising approach to addressing these issues. The present study evaluated the first implementation of a process-based diagnostic and support concept (StaFF) in everyday school practice in the Canton of Basel-Landschaft, Switzerland. Over the course of one school term, teachers (*N* = 173 at T1; *N* = 83 at T2) and pupils (*N* = 1072 at T1; *N* = 339 at T2) from kindergarten to the lower secondary level (approximately ages 4 to 16) were assessed using standardised questionnaires. Teacher outcomes included emotional exhaustion, occupational self-efficacy, subjective personal accomplishment, and attitudes towards inclusion. Pupil behaviour was assessed with the Strengths and Difficulties Questionnaire (SDQ). Data analyses comprised descriptive statistics, paired *t*-tests, and effect size estimates. The results indicated stable values for emotional exhaustion and attitudes towards inclusion, while occupational self-efficacy and perceived accomplishment significantly increased with medium to large effect sizes. At T1, more than one-third of pupils displayed at least one abnormal score; at T2, about one-third of these pupils no longer showed abnormal scores. The findings suggest that structured diagnostics combined with universal SEL measures can strengthen teachers’ professional agency and contribute to improvements in pupil outcomes while highlighting the need for long-term and multi-tiered implementation research.

## 1. Introduction

### 1.1. Problem Statement

Social-emotional and behavioural difficulties rank among the most common impairments in childhood and adolescence. International meta-analyses indicate that around 13–20% of children and young people meet criteria for a psychiatric disorder, particularly in the areas of anxiety, depression, hyperactivity, and conduct problems (Polanczyk et al., 2015; Sacco et al., 2024; Vasileva et al., 2021). Such difficulties are closely linked to educational challenges as repeating a grade, truancy and dropping out of school, reduced social participation, and an increased risk of long-term adverse outcomes in adulthood (Patel et al., 2018; Schulte-Körne, 2016). It is the school’s responsibility to support the socio-emotional development of its pupils, but internalising problems such as anxiety and depression are especially likely to be overlooked in school settings (Bilz, 2014), even though systematic reviews have established robust associations with learning difficulties and school absenteeism (Finning et al., 2019a, 2019b). Consequently, a substantial proportion of students experience difficulties that are highly relevant for both individual development and educational opportunities, yet these difficulties are not addressed by classroom intervention. At the same time, teachers tend to overestimate externalising problems (Baumgaertel et al., 1995; F. Klasen et al., 2016), which leads to resources being invested in the wrong areas with regard to students’ social-emotional development.

Teachers are likewise significantly affected by these conditions. Classroom disruption, heterogeneous student populations, and the responsibility for inclusive education—understood here as the joint teaching and support of students with and without special educational needs within regular classrooms—are among the most important stressors reported internationally (Sharma & Sokal, 2016). These are closely associated with emotional exhaustion, lower job satisfaction, and higher turnover intentions (Madigan & Kim, 2021; Skaalvik & Skaalvik, 2020). A meta-analysis by Aloe et al. (2014) further demonstrates that student misbehaviour is significantly related to all dimensions of teacher burnout, particularly emotional exhaustion but also depersonalization and personal accomplishment. Student behaviour and teacher stress are therefore closely intertwined, creating a dual challenge for inclusive education systems.

### 1.2. State of Research

Against this background, evidence-based programmes of social and emotional learning (SEL) have gained increasing importance. Recent meta-analyses underline the robust but context-dependent effects of universal, school-based SEL programmes on academic performance, well-being, and behavioural outcomes (Durlak et al., 2022). SEL strengthens students’ competencies in self-awareness, emotion regulation, empathy, and relationship skills and has been shown to improve learning outcomes and reduce problem behaviours (Durlak et al., 2011; Schulte-Körne, 2016; Taylor et al., 2017). Studies with students with special educational needs further indicate that SEL can substantially contribute to the development of core competencies and thereby enhance participation opportunities (Farmer & Adams, 2021).

Research also points to benefits for teachers. Participation in SEL-related initiatives can improve stress management, self-efficacy, and teaching quality (Jennings & Greenberg, 2009; Oliveira et al., 2021). However, the findings are not entirely consistent. Meta-analyses and reviews report heterogeneous effects that vary according to programme quality, implementation fidelity, and school context (Murano et al., 2020; Weare & Nind, 2011). Evidence also suggests that sustainable improvements in stress and burnout symptoms among teachers occur only when SEL is systematically implemented (Oliveira et al., 2021). These divergent results underscore the importance of considering contextual factors and standards of implementation.

Particularly promising is the integration of SEL into multi-tiered systems of support (MTSS). Such frameworks combine universal, selective, and indicated interventions to provide differentiated support according to student needs. Evidence indicates that this combination is especially effective, as it links preventive strategies with targeted assistance (Farmer & Adams, 2021; Oberle et al., 2016). Moreover, studies with students experiencing pronounced emotional and behavioural difficulties demonstrate that even this group can benefit from SEL when it is embedded in a structured, multi-tiered framework (Farmer & Adams, 2021).

The present study builds on this evidence. During the implementation of a standardised diagnostic tool and evidence-based preventive SEL programmes, it examines changes in central indicators at the teacher level—emotional exhaustion, occupational self-efficacy, self-rated performance, and attitudes towards inclusion—as well as at the student level—behavioural difficulties—over the course of one school semester. In doing so, it makes a dual contribution: first, by providing evidence on the effectiveness of standardised diagnostics and universal support measures under routine school conditions and second, by informing the international debate on the role of SEL and diagnostic practices in inclusive education systems.

## 2. Materials and Methods

### 2.1. Design

The study was conducted as a longitudinal intervention study under routine school conditions with two measurement points over the course of one school semester. It followed a pre–post design without randomisation, as all participating schools implemented the StaFF standards (Hövel et al., 2025) as part of their regular practice. StaFF is a structured, process-based framework that combines standardised behavioural diagnostics with guided implementation of evidence-based SEL programmes. The study was thus designed to examine how standardised diagnostic procedures and universal support measures operate under everyday school conditions.

**Figure 1:**
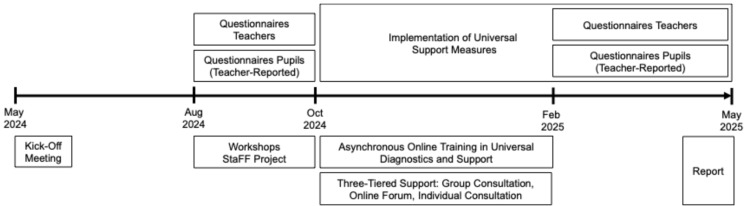
Timeline of the StaFF-BL project implementation from May 2024 to May 2025.

The research protocol was reviewed and approved by the Canton of Basel-Landschaft. All participating teachers were provided with written information and gave their consent to participate anonymously. The overall timeline of the StaFF-BL project, including key milestones and assessment phases, is shown in Figure 1.

### 2.2. Measures Implemented

Kindergarten, primary, and lower secondary schools in the Canton of Basel-Landschaft were informed about the project by the cantonal education authority and invited to participate. Within the participating schools, individual teachers decided to take part with their classes, meaning that not all school sites were fully involved. Participation was voluntary and without financial or material incentives.

In May 2024, an online kick-off meeting was held for school leaders. In summer 2024, half-day professional development workshops were conducted at the participating schools. These sessions introduced the aims, contents, and procedures of the StaFF project, presented the individual steps of the process as outlined in the project materials, and specified the project timeline. Schools were granted flexibility in adapting the implementation to their specific organisational conditions. Following the workshops, teachers were provided with access to additional information and programme materials through the digital learning platform ILIAS.

Prior to the workshops, teachers prepared digital class lists with the names of their pupils and assigned each pupil a sequential number. During registration, teachers completed an online questionnaire, programmed in LimeSurvey (https://www.limesurvey.org/de, accessed on 29 September 2025), consisting of a registration section and demographic items, alongside scales measuring theoretical constructs described below. They also reported their class size and their personal ID, a pseudonymised identification code enabling anonymous and data protection-compliant data allocation. No personal data from teachers were collected. On this basis, the research team generated unique identifiers for each pupil, consisting of the teacher’s ID and the pupil’s sequential number (e.g., et12345_01). The generated data were used to produce individualised feedback reports for teachers, which were made accessible via a secure HfH SharePoint server.

Afterwards, teachers received online questionnaires to assess all pupils in their class (baseline assessment, T1). The distribution was carried out via pseudonymised ID e-mail addresses (e.g., et12345@xy.z).

In October 2024, the implementation of universal support measures began. Originally planned until the end of 2024, due to delays in school-internal procurement of the programmes, the intervention phase was extended until spring 2025. Schools were asked to select from a range of evidence-based SEL programmes. Two criteria guided programme selection: (1) the programme had to be commercially available without prerequisites such as mandatory prior training, and (2) at least one controlled evaluation study with a positive effect had to have been published in the German-speaking context.

Based on these criteria, the following programmes (latest editions) were available: Lubo aus dem All! (Lubo from Outer Space; Kindergarten; Hillenbrand et al., 2022b; focus: all five SEL aspects), Lubo aus dem All! (Lubo from Outer Space; Grades 1–2; Hillenbrand et al., 2022a; focus: all five SEL aspects), Verhaltenstraining für Schulanfänger (Behaviour Training for School Beginners; Grades 1–2; Petermann et al., 2024; focus: all five SEL aspects), Verhaltenstraining in der Grundschule (Behaviour Training in Primary School; Grades 1–6; Petermann et al., 2019; focus: all five SEL aspects), Ben & Lee (Grades 3–4; Urban et al., 2018; focus: all five SEL aspects), Emotionstraining in der Schule (Emotion Training at School; Grades 3–6; Petermann et al., 2016; focus: self- and social-awareness and self-regulation), Fit for Life (Grades 7–9; Jugert et al., 2017; focus: all five SEL aspects), Fairplayer.Manual (Grades 7–9; Scheithauer et al., 2019; focus: self- and social-awareness and self-regulation), SNAKE—Stressbewältigung im Jugendalter (Stress Management in Adolescence; Grades 7–9; Beyer & Lohaus, 2018; focus: self- and social-awareness and self-regulation).

To support teachers during implementation, a three-tiered support structure was established: (a) regular group consultation meetings (first-level support), (b) an online discussion forum on ILIAS (second-level support), and (c) individual consultation sessions (third-level support). Group consultations were held online once per month (approximately 60 min each) and focused on current implementation progress, challenges, and exchange of good practice among teachers. The online discussion forum and the option for individual consultation were available throughout the semester but were not actively used by participants. Implementation fidelity was supported through monthly online consultation sessions in which current progress, barriers, and next steps were discussed with participating teachers. Attendance at these sessions was recorded; however, no structured fidelity checklists or teacher logs were collected, i.e., fidelity was monitored qualitatively rather than quantitatively.

In February 2025, the second assessment phase (T2) commenced. In addition to another teacher survey, pupil assessments were repeated, focusing specifically on those children who had shown at least one elevated score at T1. In these cases, teachers were asked to seek additional ratings from a second teacher or specialist and, where possible, from an external caregiver (e.g., parents), in order to obtain a multi-informant perspective. The respective data will not be considered for the following analyses, as the focus will be on the perspective of the teachers and their perception of changes in pupils’ behaviour during the period of interest.

The combination of standardised diagnostics, programme-based universal measures, and tiered consultation formed the core of the StaFF standards. A detailed description of the process, materials, and implementation logic has been published elsewhere (Hövel et al., 2025).

### 2.3. Sample

A total of eight schools (kindergarten, primary, and lower secondary level) in the Canton of Basel-Landschaft participated in the project.

At the first survey in autumn 2024 (T1), 173 teachers took part. Of these, 77.6% were female and 22.4% male. The mean age was 44.9 years (*SD* = 11.9), and the average professional experience was 14.9 years (*SD* = 10.6). The average workload was 73.9% (*SD* = 22.6). With regard to professional roles, 49.1% were class teachers, 26.6% subject teachers, 13.9% special education teachers, 0.6% school leaders, 0.6% classroom assistants, 0.6% speech therapists, 2.9% school social workers, 4.0% social pedagogues, and 1.7% other functions.

At the second survey in winter/spring 2025 (T2), 83 teachers participated. Of these, 82.7% were female and 17.3% male. The average professional experience was 17.5 years (*SD* = 9.9), and the average workload was 75.9% (*SD* = 23.0). The professional roles at T2 included 65.1% class teachers, 18.1% subject teachers, 10.8% special education teachers, 1.2% speech therapists, and 4.8% social pedagogues. School leaders, classroom assistants, and school social workers were not represented at T2. The reduction in sample size from T1 (*N* = 173) to T2 (*N* = 83) reflects the project design: while all teachers from the participating schools were invited to complete the baseline survey, only those who actively implemented the StaFF process with their classes were eligible for the follow-up. Accordingly, all inferential analyses of teacher-level outcomes (*t*-tests and effect sizes) were calculated only for this longitudinal subsample (*n* = 83) for which data from both T1 and T2 were available. No systematic differences in demographic or professional characteristics were observed between the overall T1 sample and this longitudinal group.

At the pupil level, 1072 assessments were provided by teachers at T1. At T2, 339 follow-up assessments were completed, restricted to those pupils who had shown at least one elevated value at T1. Due to data protection regulations of the Canton of Basel-Landschaft, no additional personal information about the pupils was collected.

### 2.4. Instruments

To assess teachers’ emotional exhaustion, attitudes, and professional resources, four standardised scales were used. For reasons of test economy, only selected items with high face validity were employed. All items were rated on a six-point Likert scale ranging from 1 (does not apply at all) to 6 (fully applies), and an overview of the items used is provided in Table 1.

Emotional exhaustion (MBI-ES). Emotional exhaustion was measured with three items from the Emotional Exhaustion subscale of the Maslach Burnout Inventory—Educators Survey (MBI-ES; Maslach et al., 1997; German version: Enzmann & Kleiber, 1989). Emotional exhaustion is considered the core component of burnout and has consistently shown high internal consistencies (α = 0.85–0.90) and good construct validity in teacher samples (Maslach et al., 1997, 2001).

Subjective personal accomplishment (MBI-ES). Subjective personal accomplishment was assessed with three items from the Personal Accomplishment subscale of the MBI-ES. This scale typically shows acceptable reliability (α = 0.70–0.75) and is widely used as an indicator of perceived professional competence (Maslach et al., 1997).

Occupational self-efficacy. Five items from the Occupational Self-Efficacy Scale (Schyns & von Collani, 2014) were used to capture teachers’ confidence in successfully handling job-related challenges. The original 19-item version demonstrates excellent internal consistencies (α = 0.88–0.92) and strong construct validity; shorter versions show comparable reliability (Schyns & von Collani, 2014).

Attitudes towards inclusion (EFI-L). Teachers’ attitudes towards inclusion were assessed using seven items from the Attitudes towards Inclusion Questionnaire for Teachers (EFI-L; Seifried & Heyl, 2016). Five items from the subscale Academic Support and one item each from Personal Readiness and Social Inclusion were employed. The EFI-L has been validated in large teacher samples, showing very good internal consistencies (α = 0.81–0.90) and a stable three-factor structure (Seifried & Heyl, 2016).

The selection of items was guided by content-related considerations of their relevance to the study’s research questions and practical feasibility in school settings. Items were chosen that best represented the core constructs while maintaining clarity and brevity for teachers. The psychometric quality of these abbreviated scales was subsequently examined in the present sample to verify their reliability.

The reliability coefficients (McDonald’s ω) were calculated in the present sample for all four scales (Emotional exhaustion: ω = 0.82; Subjective personal accomplishment: ω = 0.85; Occupational self-efficacy: ω = 0.84; Attitudes towards inclusion: ω = 0.89).

To assess pupils’ emotional and behavioural difficulties, the Strengths and Difficulties Questionnaire (SDQ) was administered in its German version (R. Goodman, 1997; H. Klasen et al., 2003). Teacher and parent forms for ages 4–17 were used. The SDQ consists of 25 items covering five subscales (Emotional symptoms, Hyperactivity/inattention, Conduct problems, Peer problems, Prosocial behaviour) and a total difficulties score. International studies demonstrate a stable factor structure, good discriminant validity between clinical and non-clinical populations, and strong correlations with other established measures (R. Goodman, 1997; H. Klasen et al., 2003). In German samples, the internal consistencies for the total difficulties score are typically in the acceptable to good range (α ≥ 0.80), while the brevity of the subscales often results in lower coefficients.

Beyond the five subscales, the SDQ allows the computation of internalising problems (emotional symptoms and peer problems) and externalising problems (hyperactivity/inattention and conduct problems). Research shows that these higher-order dimensions are psychometrically robust and capture the distinction between inwardly directed difficulties (e.g., anxiety, withdrawal) and outwardly directed difficulties (e.g., impulsivity, aggression) (A. Goodman et al., 2010; Stone et al., 2010). Thus, the SDQ provides a valid tool to simultaneously assess both internalising and externalising domains of child and adolescent mental health.

The reliability coefficients (McDonald’s ω) were calculated in the present sample for the total difficulties score as well as for the five subscales emotional problems (ω = 0.81), peer problems (ω = 0.78), conduct problems (ω = 0.80), hyperactivity/inattention (ω = 0.87), and prosocial skills (ω = 0.84).

### 2.5. Data Analysis

All analyses were conducted using R (version 4.4.3; R Core Team, 2025). Descriptive statistics (means, standard deviations, and percentages) were calculated to describe the sample characteristics and scale scores.

For teachers, pre–post changes between T1 and T2 were analysed using paired-samples *t*-tests. In addition, standardised effect sizes were calculated (Cohen’s *d*) to evaluate the magnitude of change.

For pupils, analyses were restricted to descriptive statistics. Specifically, the proportions of children rated as normal, borderline, or abnormal on the four SDQ problem scales (Emotional symptoms, Conduct problems, Hyperactivity/inattention, Peer problems) and on the overall index (at least one abnormal score across the four scales) were reported. Furthermore, the proportion of pupils classified as abnormal at T1 who continued to be rated as abnormal at T2 was calculated.

### 2.6. Data and Material Availability

All instruments used in this study (teacher questionnaires, SDQ forms, and SEL programme manuals) are either openly available or can be obtained from commercial publishers. The anonymised datasets generated and analysed during the current study will be deposited in the Open Science Framework (OSF) upon completion of the project. The DOI will be provided prior to publication.

## 3. Results

### 3.1. Results for Teachers

**Figure 2:**
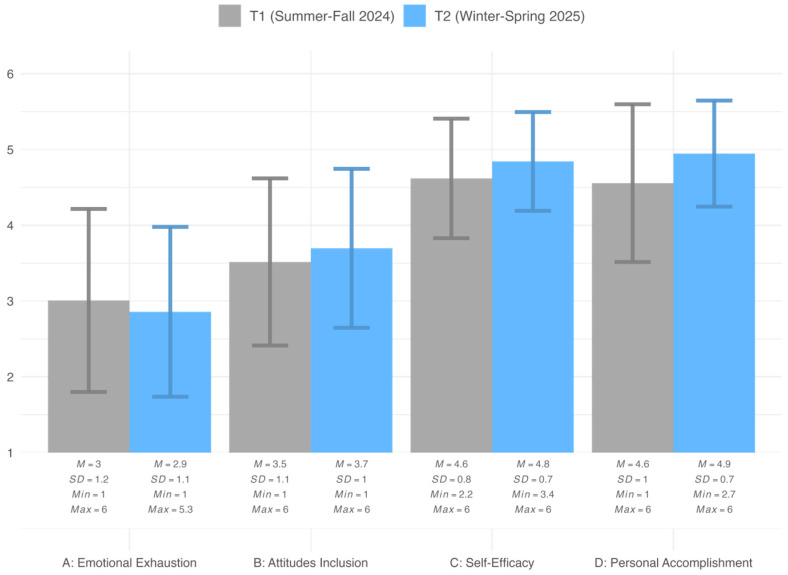
Teacher survey results at T1 and T2 (*n* = 83).

Figure 2 presents the descriptive statistics for teachers’ emotional exhaustion, attitudes towards inclusion, occupational self-efficacy, and subjective personal accomplishment at T1 and T2.

On average, emotional exhaustion at T1 was relatively low, though individual variation between teachers was substantial. At T2, mean levels were slightly lower, but this difference was not statistically significant, *t*(77) = −0.91, *p* = 0.36, with a very small effect size (*d* = −0.20).

A similar pattern emerged for attitudes towards inclusion. Scores at T1 were in the mid-range of the scale and increased only minimally at T2. The change was not statistically significant, *t*(77) = 1.40, *p* = 0.17, with a very small effect size (*d* = 0.30).

In contrast, occupational self-efficacy was already high at T1 and showed a significant increase at T2, *t*(78) = 2.72, *p* = 0.01, corresponding to a medium-to-large effect (*d* = 0.59). Similarly, subjective personal accomplishment was high at T1 and increased significantly from T1 to T2, *t*(78) = 3.10, *p* < 0.001, with a medium-to-large effect (*d* = 0.68).

### 3.2. Results for Pupils

**Figure 3:**
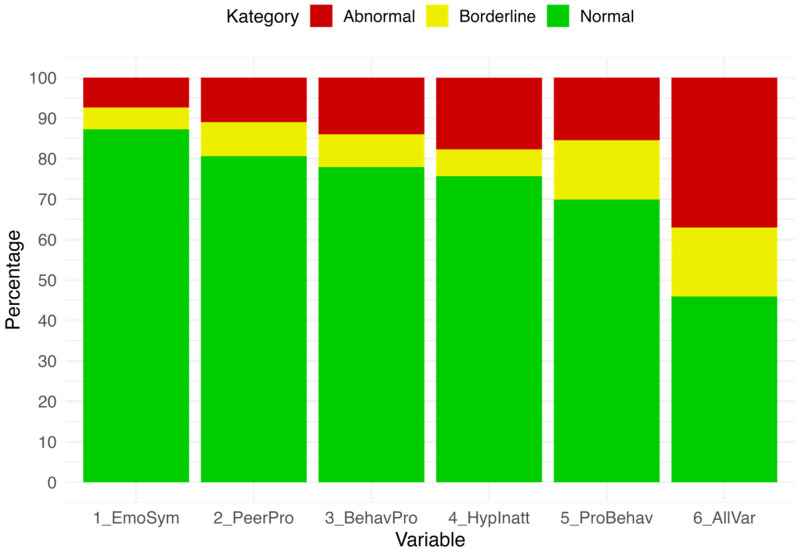
Distribution of SDQ teacher ratings at T1 (Summer–Autumn 2024; n = 1072).

At T1 (Summer–Autumn 2024), a total of 1072 SDQ ratings by teachers were available. Figure 3 presents the proportions of pupils classified as normal, borderline, or abnormal across the five SDQ scales. From the teachers’ perspective, the highest rate of abnormal scores was found for Hyperactivity/Inattention (17.72%), while the lowest was observed for Emotional Symptoms (7.37%). When borderline and abnormal scores were combined, Prosocial Behaviour stood out, with 30.14% of pupils rated in these categories. Overall, 37.03% of pupils had at least one abnormal score on one of the scales and were therefore scheduled for reassessment at T2, while a further 17.07% were rated in the borderline range.

**Figure 4:**
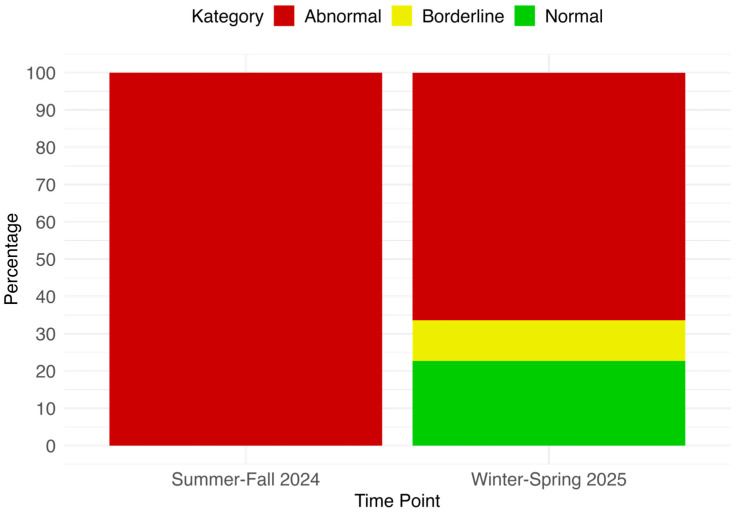
Comparison of SDQ teacher ratings for pupils classified as abnormal at T1 and re-assessed at T2 (Winter–Spring 2025; n = 339).

At T2 (Winter–Spring 2025), SDQ ratings were available for 339 pupils who had shown at least one abnormal score at T1. Figure 4 illustrates the comparison of overall abnormality between T1 and T2. All improvement percentages (e.g., 33.6%) refer to ratings provided by the same class teacher at both measurement points. Thus, changes represent within-rater comparisons rather than differences between independent informants. The results indicate a reduction in abnormal classifications: 22.71% of pupils previously rated as abnormal were no longer rated as abnormal on any SDQ scale at T2, and an additional 10.91% were classified as borderline only. In total, 33.62% of the originally abnormal cases were no longer rated as abnormal at T2 by the same teacher.

## 4. Discussion

The findings from the first implementation of the StaFF project provide initial empirical evidence on the feasibility and potential effects of a standardised, process-based diagnostic support concept in everyday school practice. Consistent with the theoretical framework introduced earlier, the results reflect how structured diagnostics and SEL-based support can affect teacher well-being and professional agency. At the teacher level, emotional exhaustion and attitudes towards inclusion remained largely stable over the course of half a school year, showing only minimal and non-significant changes. In contrast, both occupational self-efficacy and subjective personal accomplishment increased significantly, with medium to large effect sizes. These results suggest that teachers’ perceived competence and agency improved during the project. Feedback from participating teachers suggests that the structured diagnostic process and accompanying support measures may have contributed to increased confidence and reflexivity.

At the pupil level, differentiated results emerged. From the teachers’ perspective, a high proportion of abnormal SDQ scores was observed at T1, particularly for hyperactivity/inattention, while emotional symptoms showed the lowest rates. More than one-third of pupils displayed at least one abnormal score and were therefore reassessed at T2. Within this group, about one-third showed a reduction in difficulties and were no longer rated as abnormal by the same teacher. These findings provide cautious evidence of positive behavioural change in some pupils, although a substantial proportion continued to present difficulties. This underlines the need for continuous and long-term monitoring.

Taken together, the findings suggest that the StaFF project can strengthen teachers’ professional agency while at the same time fostering initial positive developments at the pupil level. However, the results also indicate that changes in deeper-seated attitudes (e.g., towards inclusion) or in teachers’ burden (e.g., emotional exhaustion) require more time and stable conditions to unfold. Previous research indicates that diagnostic insights are most effective when complemented by practice-oriented, context-sensitive interventions embedded in school routines (e.g., Farmer & Adams, 2021).

### 4.1. Interpretation in Light of Previous Research

The findings of the present study are broadly consistent with international evidence on universal, school-based SEL programmes. At the teacher level, significant increases in occupational self-efficacy and subjective personal accomplishment align with studies demonstrating that SEL-related approaches—either through classroom-based curricula or teacher-focused interventions—can strengthen teachers’ sense of competence and alleviate work-related strain. Jennings et al. (2017) reported that the CARE for Teachers programme enhanced teachers’ social-emotional competences and classroom interactions, while Oliveira et al. (2021) documented improvements in well-being and SEL resources among teachers following a mindfulness-based intervention. Evidence from routine practice further suggests that universal SEL curricula can improve the quality of teacher–student interactions and buffer against the negative effects of teacher stress (Sandilos et al., 2023).

In contrast, emotional exhaustion and attitudes towards inclusion remained stable in our sample across the half-year implementation period. This finding is plausible in light of prior work: meta-analytic evidence consistently shows small-to-moderate overall effects of SEL programmes while also emphasising dose- and implementation-related contingencies (Durlak et al., 2022; Taylor et al., 2017). More deeply rooted orientations (e.g., attitudes towards inclusion) and indicators of strain (e.g., exhaustion) may be less responsive to short-term interventions and require sustained exposure and supportive conditions over time (Jennings et al., 2017; Skaalvik & Skaalvik, 2020).

At the pupil level, our results—high rates of abnormal SDQ scores at T1, particularly in hyperactivity and prosocial behaviour, and improvements in about one-third of initially abnormal cases at T2—mirror international evidence. Improvements were more frequent in externalising domains (e.g., hyperactivity, conduct problems) than in internalising symptoms (e.g., emotional problems, peer difficulties), which aligns with previous findings that SEL interventions more readily affect observable behavioural regulation than internal emotional states (Durlak et al., 2011). Meta-analyses confirm that universal SEL programmes can reduce both externalising problems (e.g., aggression, disruptive behaviour) and internalising symptoms (e.g., anxiety, depression) (Ağırkan & Ergene, 2022; Taylor et al., 2017). Importantly, Taylor et al. (2017) also demonstrated that such benefits can persist for several years, highlighting the long-term potential of universal SEL. Recent reviews additionally show that while average effects are robust, outcomes vary depending on programme type, age group, and implementation quality (Durlak et al., 2022).

Finally, implementation quality emerges as a critical factor. Studies under routine conditions demonstrate strong associations between fidelity, classroom quality, school leadership support, and collegial collaboration, and the outcomes achieved (Neugebauer et al., 2023; Sandilos et al., 2023). These findings resonate with our observations, where teachers emphasised the importance of leadership engagement, structured diagnostic processes, and supportive consultation for successful implementation.

In sum, the present study adds to a growing body of evidence suggesting that combining diagnostic clarity with universal SEL measures can strengthen teachers’ professional agency and foster positive pupil development in inclusive educational contexts. The strength of these effects, however, depends strongly on time, context, and implementation conditions.

### 4.2. Methodological Considerations and Limitations

Several methodological issues need to be considered when interpreting the findings. First, the sample represents a self-selected group of schools and teachers. Participation in the project was voluntary, which may have led to a bias in favour of particularly motivated or innovative schools. This self-selection bias likely reflects higher-than-average engagement and openness towards inclusive and preventive approaches, which could have positively influenced both implementation quality and outcome perception. Moreover, while some schools involved entire teaching staffs, others participated only with individual classes or teachers. This limits comparability across sites and reduces the generalisability of the results.

Second, the study did not include a control group. Changes observed between T1 and T2 therefore cannot be unequivocally attributed to the project measures but may also reflect external influences such as individual developmental trajectories, school-level changes, or temporary stressors. Accordingly, the positive developments reported here should be interpreted with caution.

Third, the heterogeneity of the support programmes must be taken into account. Although only evidence-based SEL programmes were eligible, schools were able to choose from a selection of different curricula. As a result, it is not possible to disentangle the effects of specific programmes, and the intensity of implementation likely varied between schools.

Another consideration concerns the measurement instruments. The teacher questionnaires were based on abbreviated scales (emotional exhaustion, occupational self-efficacy, subjective personal accomplishment, and attitudes towards inclusion). Despite these reductions, the scales demonstrated good to excellent reliability in our sample, supporting their psychometric adequacy. The use of standardised instruments also enhanced comparability and allowed for a robust analysis of the core constructs.

For pupils, behaviour was assessed using the SDQ. This internationally established tool is validated to capture both internalising difficulties and externalising difficulties. A methodological challenge at T2 was the distinction between first and second ratings: some teachers mistakenly identified themselves as “secondary raters”, although they had already assessed the same pupils at T1. This misunderstanding may have affected data classification in some cases and will be addressed in future iterations by clarifying terminology.

Moreover, formal fidelity metrics (e.g., structured checklists or session-by-session logs) were not collected; fidelity was monitored qualitatively through monthly consultation sessions. This constrains the precision of inferences about dosage and adherence.

Finally, the time frame of the study should be noted. Data collection covered a period of only half a school year, thus reflecting short-term developments. As such, the findings provide initial rather than sustained evidence, and no conclusions can yet be drawn regarding the long-term stability or durability of observed changes. More fundamental changes in attitudes or enduring behavioural patterns are likely to require longer observation periods and more sustained structures in order to be reliably assessed.

Taken together, the findings allow for a differentiated appraisal of the first implementation under routine conditions while also highlighting the need for future evaluations with larger and more systematically composed samples, clearly defined assessment roles, and extended observation periods to draw robust conclusions on the effectiveness and sustainability of the StaFF approach.

### 4.3. Practical Implications

The findings of this project provide important guidance for the design of support structures in everyday school practice. First, the results show that standardised diagnostics in combination with evidence-based SEL programmes can strengthen teachers’ professional agency and enable the early identification of behavioural difficulties among pupils. The quantitative results show that increases in self-efficacy and perceived personal accomplishment may reflect the benefits of a clearly structured process that reduces uncertainty and fosters reflection on practice. For example, schools could integrate the StaFF process into their annual professional-development cycles by linking the diagnostic phase with regular team reflections or school-based coaching sessions, allowing staff to jointly review class data, discuss intervention strategies, and plan next steps within existing development meetings.

Second, the findings underline the importance of school-level conditions. Previous studies have emphasised that leadership support, collegial collaboration, and clear communication structures are decisive for successful implementation, which cannot depend solely on individual teachers but requires a school-wide understanding of prevention and support (e.g., Dowling & Barry, 2020; Wigelsworth et al., 2010).

Third, the pupil findings point to the potential of universal SEL measures to act preventively within regular classroom instruction. At the same time, the persistent proportion of abnormal cases suggests that universal measures alone are not sufficient. Instead, a multi-tiered system of support is needed, combining universal, selective, and indicated measures to provide differentiated support according to pupils’ needs.

Finally, several practical implications can be derived from the project experience: the importance of early integration into school year planning, the need for accessible and user-friendly digital platforms with accompanying consultation, and the value of systematically involving parents to strengthen the alignment of school-based measures. Overall, the findings underline that diagnostic procedures can only unfold their potential when they are consistently linked to practice-oriented interventions and embedded in sustainable school structures. For practice, this implies that investment in systematic professional development, leadership engagement, and multi-professional collaboration is essential to fully leverage the benefits of SEL and standardised diagnostics.

### 4.4. Future Research Directions

The first implementation of the StaFF project provides valuable insights into the feasibility and potential effects of a standardised, process-based diagnostic support concept in everyday school practice. At the same time, it highlights key areas for future research that should be addressed in subsequent studies.

First, longer observation periods are needed. Many processes—such as changes in teachers’ attitudes towards inclusion or their experiences of strain—are likely to unfold only over several school years. Longitudinal studies are therefore required to assess the sustainability of the observed effects and to capture potential long-term outcomes.

Second, future research should include more systematically composed samples. Larger and more representative groups of schools and teachers will allow for more robust conclusions and enhance the generalisability of the findings. In addition, a control group is necessary to disentangle causal effects from contextual influences.

Third, the extension to selective and indicated interventions appears particularly promising. While the first project phase focused on universal measures, future work should examine how targeted interventions for highly burdened pupils can be systematically linked to diagnostic findings. This also calls for closer multi-professional collaboration with school social work, special education, and psychological services.

Fourth, there is a need for a broader assessment of contextual variables. Both child-related factors (e.g., family stress, transition situations) and school-level conditions (e.g., team culture, leadership engagement, resources) should be considered in order to enable more differentiated analyses of mechanisms and facilitating factors.

Finally, refinements in instruments and data collection procedures require particular attention. This includes clarifying the roles of primary versus secondary raters at follow-up assessments, as well as designing digital platforms for teachers and parents to be more user-friendly and accessible.

Overall, the second project phase offers the opportunity to deepen the insights gained so far, to further institutionalise the implementation process, and to systematically evaluate the effectiveness of a multi-tiered diagnostic support approach across diverse school contexts.

In addition to the quantitative results presented here, qualitative interviews with participating teachers were conducted, focusing on their experiences with the SEL programmes, the use of behavioural diagnostics, and the provided support structures. These findings will be reported in a separate publication and can inform future research on implementation processes and contextual mechanisms.

## 5. Conclusions

This study provides initial evidence on the implementation of a standardised, process-based diagnostic concept (StaFF) under routine conditions in inclusive schools. At the teacher level, significant gains in occupational self-efficacy and perceived personal accomplishment were observed, while emotional exhaustion and attitudes towards inclusion remained stable. At the pupil level, about one-third of initially abnormal cases showed improvement over one school term. The novelty of this work lies in combining structured diagnostic procedures with universal SEL implementation under real-world school conditions—an approach rarely examined in previous research. While contextual and structural factors such as leadership and collaboration proved essential for successful implementation, the short observation period and self-selection of schools limit the generalisability of the findings.

Future research should extend the observation period and test selective and indicated interventions within the same framework.

## Figures and Tables

**Table 1:** Overview of the items used.

Scale	Item Wording (English)/(German)
Emotional exhaustion (MBI-ES; Enzmann & Kleiber, 1989; Maslach et al., 1997)	I feel emotionally drained from my work./Ich fühle mich durch meine Arbeit emotional erschöpft. I feel used up at the end of the workday./Am Ende eines Arbeitstages fühle ich mich ausgelaugt. I feel burnt out from my work with pupils./Ich fühle mich durch die Arbeit mit den Schüler:innen ausgebrannt.
Subjective personal accomplishment (MBI-ES; Enzmann & Kleiber, 1989; Maslach et al., 1997)	I feel that I am positively influencing other people’s lives through my work./Ich habe das Gefühl, durch meine Arbeit Positives zu bewirken. I feel that I am reaching pupils effectively through my work./Ich habe den Eindruck, die Schüler:innen durch meine Arbeit zu erreichen. I feel competent in my job./Ich fühle mich in meinem Beruf kompetent.
Occupational self-efficacy (Schyns & von Collani, 2014)	Even when problems arise unexpectedly, I always know how to deal with them./Auch in schwierigen Situationen weiß ich, wie ich mich verhalten sollte. I am confident that I can handle job-related challenges successfully./Ich bin zuversichtlich, berufliche Herausforderungen erfolgreich zu meistern. When unexpected problems occur, I can find solutions./Wenn unerwartete Probleme auftreten, finde ich Lösungen. I possess the skills necessary for professional success./Ich verfüge über die Fähigkeiten, die für beruflichen Erfolg notwendig sind. I know that I can cope with the demands in my job./Ich weiß, dass ich mit den Anforderungen in meinem Beruf umgehen kann.
Attitudes towards inclusion (EFI-L; Seifried & Heyl, 2016)	Pupils with behavioural difficulties can be taught as effectively in subject lessons as other pupils./Schüler:innen mit Verhaltensauffälligkeiten können im Fachunterricht gleich gut gefördert werden wie andere. I am willing to include pupils with challenging behaviour in my teaching./Ich bin bereit, Schüler:innen mit herausforderndem Verhalten in meinen Unterricht einzubeziehen. In my class, pupils with special needs can be socially included./In meiner Klasse können Schüler:innen mit Förderbedarf sozial integriert werden.

## Data Availability

The original data presented in the study are openly available the Open Science Framework (OSF) repository at https://doi.org/10.17605/OSF.IO/4DYJ5 (accessed on 1 July 2025).

## Abbreviations

The following abbreviations are used in this manuscript:

SEL	Social and Emotional Learning
StaFF	Standards for the Assessment of Support Needs and Universal Support
SDQ	Strengths and Difficulties Questionnaire
EFI-L	Questionnaire on Attitudes towards Inclusion for Teachers
MBI-ES	Maslach Burnout Inventory—Educators Survey
OSF	Open Science Framework
ICMJE	International Committee of Medical Journal Editors
